# Associations of Dietary Bioactive Compounds with Maternal Adiposity and Inflammation in Gestational Diabetes: An Update on Observational and Clinical Studies

**DOI:** 10.3390/ijerph17207528

**Published:** 2020-10-16

**Authors:** Dustin W. Davis, Jeannette Crew, Petar Planinic, James M. Alexander, Arpita Basu

**Affiliations:** 1 Department of Kinesiology and Nutrition Sciences, University of Nevada, Las Vegas, NV 89154, USA; dustin.davis@unlv.edu (D.W.D.); crewj1@unlv.nevada.edu (J.C.); 2 Department of Obstetrics & Gynecology, School of Medicine, University of Nevada at Las Vegas, Las Vegas, NV 89154, USA; pplaninic@gmail.com (P.P.); james.alexander@unlv.edu (J.M.A.)

**Keywords:** gestational diabetes, inflammation, diet therapy, vitamins, minerals, omega-3 fatty acids, probiotics, synbiotics, trace elements

## Abstract

Gestational diabetes mellitus (GDM) is a common complication of pregnancy that adversely affects maternal and offspring health. Maternal obesity, oxidative stress, and inflammation have been implicated in GDM. In non-pregnant adults, intakes of dietary bioactive compounds inversely associate with insulin resistance and inflammation. However, associations of dietary bioactive compounds with biomarkers of adiposity, antioxidant vitamin and mineral status, oxidative stress, and inflammation in GDM have not been fully elucidated. We addressed this gap by conducting a semi-quantitative review of observational studies and randomized controlled trials published between 2010 and 2020 and retrieved from Google Scholar, Medline, and PubMed. Our analysis revealed that women with GDM are more likely to consume a pro-inflammatory diet before pregnancy and tend to consume fewer antioxidant vitamins and minerals during pregnancy than healthy pregnant women. Women with GDM also have lower blood levels of vitamins A, C, and D and certain adipokines. Several dietary bioactive compounds were noted to improve antioxidant status and biomarkers of inflammation. The Dietary Approaches to Stop Hypertension (DASH) diet and soybean oligosaccharides increased antioxidant enzyme levels. Supplementing *n*-3 fatty acids, probiotics, synbiotics, and trace elements increased antioxidant enzymes and reduced hs-CRP and MDA. Improvements in inflammation by vitamin D may be contingent upon co-supplementation with other dietary bioactive compounds.

## 1. Introduction

Gestational diabetes mellitus (GDM) is characterized by the onset of hyperglycemia during pregnancy, typically in the second trimester, and is the most prevalent metabolic complication in pregnancy globally [1]. Diagnostic criteria differ by region and are largely influenced by conventional care and the preferences of the clinicians. The lack of uniformity in diagnosing GDM makes it difficult to accurately estimate its global prevalence. However, recent reviews concluded that GDM is most prevalent in the Middle East and North Africa (15.2%, 8.8–20.0% [median, interquartile range]) and South-East Asia (15.0%, 9.6–18.3%). The prevalence is lowest in North America and the Caribbean (7.0%, 6.5–11.9%) and Europe (6.1%, 1.8–31.0%), though the rates among European countries vary widely [1,2].

Increases in maternal blood glucose with advancing pregnancy, and glucose intolerance during gestation, even within the normal range, accompany a graded increase in the risk of maternal and fetal complications before and around birth [3,4]. Fasting and postprandial hyperglycemia, as occurs in GDM, are strongly and continuously associated with higher odds of elevated cord-blood serum C-peptide (a marker of inflammation), pre-eclampsia, preterm and primary cesarean delivery, shoulder dystocia, infants who are large for gestational age, neonatal hypoglycemia, and admitting newborns to a neonatal intensive care unit [5]. While acute risks to immediate maternal and neonatal health typically resolve shortly after delivery, both the mother and infant often experience long-term consequences associated with GDM. Compared to mothers with normoglycemia during pregnancy, mothers with GDM suffer a greater-than-sevenfold increase in the risk of developing type 2 diabetes mellitus later in life (T2DM, relative risk = 7.43) [6]; consequently, GDM is the preeminent predictor of T2DM [7]. Further, retrospective estimates in parous women with diabetes mellitus suggest that 10–31% of cases were diagnosed with GDM [7]. In addition to this postpartum risk in humans, animal models suggest that the fetuses of dams with GDM are metabolically programmed in a diabetic milieu, raising the risk of the offspring developing obesity, hyperglycemia, T2DM, and premature cardiovascular disease after birth [1,8]. A follow-up study with the adult children of women with GDM showed that 21% had prediabetes or T2DM, indicating an eight-fold-greater risk of T2DM than in the children of women deemed to have a low genetic risk of T2DM [9]. Insulin secretion and sensitivity were also impaired, and the prevalence of overweight and metabolic syndrome were two- and four-fold greater, respectively [10]. Increased adiposity, fasting blood glucose, insulin resistance, and overall cardiovascular risk profile have also been observed in the offspring of mothers with GDM [11,12,13,14].

Non-modifiable risks of GDM include genetics, ethnicity, and advancing maternal age [1]. Of chief importance among modifiable risks are physical activity and dietary intake before and after conception [1,15,16]. The reduced level of physical activity during pregnancy is partly responsible for the pregnancy-associated decline in metabolic health [17,18]. Suboptimal maternal nutrition is also common, especially in Western countries such as the United States (US). Pregnant US women over-consume sodium and iron and under-consume the antioxidant vitamins C and E [19], potentially exacerbating gestational oxidative stress and inflammation. Inadequate physical activity and poor nutrition collectively contribute to overweight (body mass index [BMI] ≥ 25 kg/meter^2^ [kg/m^2^]) and obesity (BMI ≥ 30.0 kg/m^2^). Critically, having overweight or obesity is the strongest predictor of GDM [16], and obesity has been concretely established as a mediator of chronic, low-grade, systemic inflammation [20,21]. While prenatal lifestyle counseling has not been definitively shown to reduce excessive gestational weight gain [22], the actual implementation of lifestyle modifications reduces the risk of GDM, especially with early initiation before 15 weeks of pregnancy [23,24].

Modifying the diet is a key target in the prevention and treatment of GDM. Data about the effects of specific nutrients on GDM risk come from cross-sectional or retrospective studies with small numbers of GDM cases, thus precluding definitive conclusions on cause and effect [16]. Nonetheless, several studies have linked diets low in fiber [25] and high in sugar-sweetened soda [26]; potatoes [27]; fried foods purchased outside of the home [28]; red and processed meat [29]; fat [30], cholesterol [30], and protein from animals [31]; and heme iron [32] (all characteristic of a Western dietary pattern) with a heightened risk of GDM. Inversely, greater intakes of fiber [25,33], fruits [33], green leafy vegetables [33], and protein from nuts and lean animal proteins such as poultry and fish [31] (all characteristic of the Mediterranean diet) associate with reductions in the risk of GDM. Data on the effects of specific dietary bioactive compounds in foods were reviewed in 2016 [34], and more evidence that deserves attention has since accumulated. For example, the interest in dietary polyphenols, a type of dietary bioactive compound, is growing. Dietary polyphenols have improved insulin resistance in a rat model of GDM [35] and, in humans, were associated with a reduced risk of GDM [36]. Overall, available data suggest a promising role of dietary bioactive compounds in reducing the risk of GDM. What remains to be elucidated is how these compounds impact biomarkers of adiposity, antioxidant vitamin and mineral status, oxidative stress, and inflammation in women who already have GDM.

The age-standardized global obesity rate among adult women rose from 6.4 to 14.9% between 1975 and 2014 [37]. The rate rose further since, disproportionately so in certain countries such as the US, where 41.9 and 11.5% of women aged ≥ 20 years had obesity and severe obesity (BMI ≥ 40.0 kg/m^2^), respectively, in 2018 [38]. Mitigating the progression of obesity and inflammation to reduce adverse consequences during pregnancies, especially those affected by GDM, must involve dietary modification. As previously stated, many studies have identified foods and nutrients linked to a lower risk of GDM [25,31,33]. Yet data on the role of dietary bioactive compounds, such as polyphenols, antioxidant vitamins, omega-3-fatty acids, pre- and probiotics, and synbiotics, in improving adiposity, antioxidant vitamin and mineral status, oxidative stress, and inflammation in GDM are comparatively scarce. Thus, we conducted the present review to synthesize and describe the latest data from observational studies and randomized controlled trials (RCTs) on the effects of dietary bioactive compounds on biomarkers of maternal adiposity, antioxidant vitamin and mineral status, oxidative stress, and inflammation in GDM.

## 2. Materials and Methods

**Figure 1:**
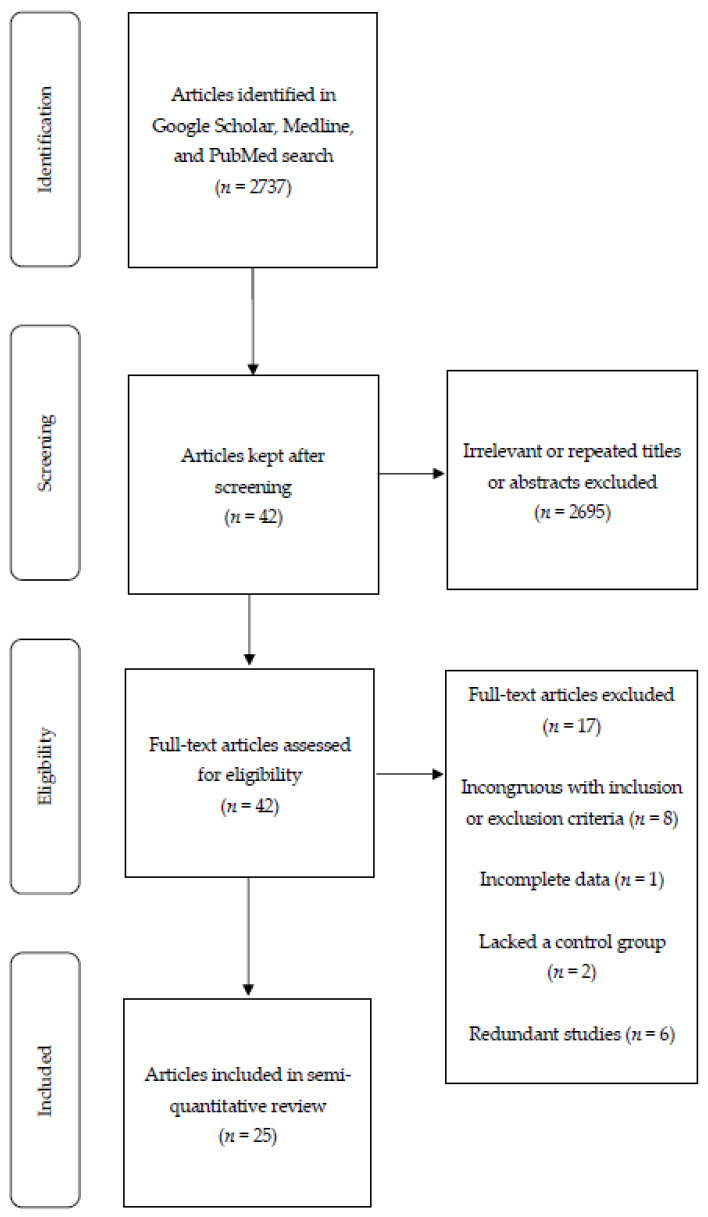
Flow diagram depicting the search and selection of published articles using our inclusion and exclusion criteria.

The literature search focused primarily on observational studies and RCTs investigating the role of dietary bioactive compounds on maternal biomarkers of adiposity, antioxidant vitamin and mineral status, oxidative stress, and inflammation in women with GDM. Searches were conducted utilizing the online databases Google Scholar, Medline, and PubMed. Selected articles were limited to those published from 2010 to 2020. Search keywords included “gestational diabetes,” “oxidative stress,” “inflammation,” “diet,” “nutrition,” “dietary bioactive compounds,” “supplements,” “polyphenols,” “flavonoids,” “vitamins,” “minerals,” “tea,” chocolate,” “nuts,” “whole grain,” “oil,” “fruits,” “vegetables,” “dairy,” “meat,” “plant-based,” “DASH,” “Mediterranean,” “prebiotics,” and “probiotics.” This search resulted in a total of 2737 articles (Figure 1), which were assessed by title first and abstract second as per our predetermined eligibility criteria. Articles whose title and abstract matched these criteria were then assessed again with the same criteria by examining their full texts. Ultimately, 25 articles reporting 24 studies were eligible and included. The discrepancy between the number of articles and the number of studies exists because two articles were published on one of the RCTs (Table 2) [39,40].

Eligibility was determined as follows. The inclusion criteria for observational studies were a comparison of women with GDM with healthy pregnant controls, the measurement of dietary bioactive compounds consumed or their metabolites in maternal blood, and the measurement of biomarkers of maternal adiposity, antioxidant vitamin and mineral status, oxidative stress, and/or inflammation in vivo. The inclusion criteria for RCTs were studies involving women with GDM, a dietary intervention with a randomized assignment of participants to the experimental and control/placebo groups, and the measurement of biomarkers of maternal adiposity, antioxidant vitamin and mineral status, oxidative stress, and/or inflammation in vivo. The exclusion criteria for both observational studies and RCTs were non-human models, not including women with GDM, the lack of a control/placebo group, a dietary intervention administered outside of the gestational period, maternal biomarkers outside of the gestational period, and a lack of data on maternal biomarkers. J.C. conducted the search and compiled the articles for data collection. D.W.D. and A.B. assisted with the search and verified that the selected articles aligned with the eligibility criteria.

For our semi-quantitative analysis, we reported means and calculated standard deviations and percentage differences for outcome measures using the data reported in the results sections of the articles that we included. Standard errors of the mean (SE) were converted to standard deviations (SD) using the formula SD = SE x √*n*, where *n* was the sample size, so that all results are reported consistently. Results that are not expressed as percentage differences are stated as otherwise.

## 3. Results

### 3.1. Observational Studies

The seven observational studies [41,42,43,44,45,46,47] were conducted in Canada [44], India [47], Iran [41,42,43,45], and South Korea [46] (Table 1). Five studies had a case-control design [41,42,44,45,46] and two had a cross-sectional design [43,46]. In all of the studies except one [46], the mean age in years of women with GDM was in the twenties or early thirties. Two studies included women with normal weight and GDM (one of those two studies also included women with overweight and GDM) [46,47]; the five other studies included women with overweight and GDM [41,42,43,44,45]. None of the studies involved women with obesity and GDM. Six of the studies compared women with GDM with healthy pregnant controls [41,42,43,44,45,46], and one study compared women with GDM with both healthy pregnant and healthy non-pregnant controls [47].

#### 3.1.1. Nutrient Intake and Risk of GDM

Three studies used food frequency questionnaires (FFQ) to determine nutrient intake and evaluate participants’ GDM risk [41,42]; total antioxidant capacity (TAC) of blood [42]; and biomarkers of adiposity and inflammation, including adipokines and C-reactive protein (CRP) [46]. Using a 147-item FFQ and the dietary inflammatory index (DII), Shivappa et al. (2019) calculated the degree to which participants’ diets were inflammatory in the year preceding pregnancy (higher scores indicated a pro-inflammatory diet) [41]. Compared to participants in the first tertile of DII scores, participants in the third tertile had double the risk of GDM after adjusting for relevant covariates (*p* = 0.03) [41]. Parast and Paknahad (2017) utilized a 168-item FFQ and reported lower intakes of vitamin E (−37%, *p* < 0.001), selenium (−17%, *p* = 0.037), and zinc (−31%, *p* < 0.001) but not β-carotene and vitamin C among women with GDM compared to healthy pregnant controls [42]. Additionally, the TAC of women with GDM was 61% lower (*p* < 0.001) [42]. For each unit increase in TAC (μmol/L), the odds of GDM decreased by nearly 10% (*p* < 0.001) [42]. In the third and final study using an FFQ, Park et al. (2013) reported that women with GDM consumed more energy, carbohydrates, fiber, and fat than healthy pregnant controls (*p* < 0.05) [46].

#### 3.1.2. Biomarkers of Adiposity, Antioxidant Vitamin and Mineral Status, Oxidative Stress, and Inflammation

Five studies compared the concentrations of biomarkers of adiposity, antioxidant vitamin and mineral status, oxidative stress, and/or inflammation between women with GDM and healthy pregnant controls [43,44,45,46,47]. Haidari et al. (2016) and McManus et al. (2014) reported blood 25-hydroxyvitamin D (25(OH)D) to be 26% (*p* = 0.034) [43] and 21% (*p* = 0.01) [44] lower, respectively, in women with GDM compared to the controls. Haidari et al. (2016) also reported that neither high-sensitivity-CRP (hs-CRP) nor tumor necrosis factor-alpha (TNF-α) differed between the groups [43]. McManus et al. (2014) also reported lower adiponectin (−95%, *p* = 0.002) and resistin (−26%, *p* = 0.045) in GDM compared to the control group, but inflammatory biomarkers did not differ [44]. In one of the three other observational studies, Javadian et al. (2014) reported ferritin and malondialdehyde (MDA) to be 26% (*p* = 0.012) and 114% (*p* = 0.0001) higher, respectively, in women with GDM compared to the control group [45]. Separately, Park et al. (2013) reported women with overweight and GDM to have 42% lower adiponectin than pregnant controls with overweight but not GDM (*p* < 0.05) [46]. Visfatin was lower and CRP was higher in women with overweight and GDM compared to pregnant women with normal weight and without GDM (visfatin −119% and CRP +125%, *p* < 0.05) [46]. Lastly, Suhail et al. (2010) reported MDA to be 14% higher (*p* = 0.001) and vitamins A, C, and E to be 13% (*p* = 0.012), 21% (*p* = 0.025), and 34% (*p* < 0.001) lower in women with GDM than in the control group [47].

### 3.2. Randomized Controlled Trials

The 17 RCTs originated from China [48,49], Iran [39,40,50,51,52,53,54,55,56,57,58,59,60,61,62], and the USA [63] (Table 2). Fourteen of the RCTs [39,40,48,50,51,52,53,54,55,56,57,58,59,60,62] were double blinded, and three were not [49,61,63]. Women were aged in their twenties [39,40,48,50,51,52,54,55,56,58,60,61,62,63] and thirties [53,57,59] in 13 and three trials, respectively. Eleven trials included women with overweight and GDM [50,51,52,53,54,55,56,57,58,60,61], and five trials included women with obesity and GDM [39,40,48,59,62,63]. The one remaining trial reported that women were over 20 years of age but did not report the mean or SD for age or BMI [49].

#### 3.2.1. Diet Therapy

Two RCTs measured the effects of adjusting macronutrient intake on biomarkers of adiposity and/or antioxidant status in women with GDM. Hernandez et al. (2016) compared a diet high in complex carbohydrates and low in fat (CHOICE) to a conventionally recommended diet low in carbohydrates and high in fat (LC/CONV). After the intervention, oxidized LDL (OxLDL) did not significantly differ based on the diet [63]. Asemi et al. (2013) reported that the Dietary Approaches to Stop Hypertension (DASH) diet did not affect hs-CRP but increased TAC and glutathione (GSH) by 6% (*p* < 0.0001) and 12% (*p* < 0.0001), respectively [62]. In addition to the CHOICE and DASH diets, soy-based diets have been investigated as a therapy for inflammation in GDM [49,61]. Jamilian and Asemi (2015) reported that MDA decreased by 3% with a soy-based diet (*p* = 0.04; adjusted change = −0.2 ± 0.2 μmol/L, *p* = 0.04) but that changes in other biomarkers of antioxidant defenses and inflammation did not differ [61]. Separately, Fei et al. (2014) provided women with GDM with soybean oligosaccharides and insulin treatment compared to insulin alone. Compared to the placebo group at eight weeks, the group receiving soybean oligosaccharides had levels of adiponectin, catalase, glutathione peroxidase (GPx), and superoxide dismutase that were 94% (*p* < 0.01), 46% (*p* < 0.01), 21% (*p* < 0.01), and 22% (*p* < 0.01) greater, respectively [49]. Additionally, the soybean oligosaccharide group had 53% lower thiobarbituric acid reactive substances (TBARS) after treatment (*p* < 0.01) [49].

#### 3.2.2. Omega-3 (*n*-3) Fatty Acids

Three RCTs supplemented *n*-3 fatty acids in women with GDM [50,53,56]. Jamilian et al. (2020) reported that *n*-3 fatty acids from flaxseed oil containing alpha-linolenic acid decreased hs-CRP (β = −1.27, *p* = 0.006) and MDA (β = −0.47, *p* < 0.001), and increased GSH (β = +116.55, *p* = 0.006) and total nitrite (β = +5.42, *p* < 0.001) compared to placebo group [50]. Similarly, Jamilian et al. (2018) reported that fish oil containing docosapentaenoic acid (DHA) and eicosapentaenoic acid (EPA) decreased hs-CRP by 49% (*p* = 0.01) [53]. In a third RCT, Razavi et al. (2017) reported that giving *n*-3 fatty acids (DHA and EPA) concomitantly with vitamin D decreased hs-CRP and MDA by 24% (*p* = 0.008; adjusted change −1.9 ± 0.5 mg/L) and 17% (*p* < 0.001; adjusted change—0.5 ± 0.1 μmol/L), respectively [56]. The intervention also increased GSH and TAC by 15% (*p* < 0.001) and 10% (*p* < 0.001), respectively [56]. The adjusted increase in NO was significant (+1.0 ± 1.6 μmol/L, *p* = 0.02).

#### 3.2.3. Probiotics and Synbiotics

Three RCTs provided probiotics to women with GDM [39,40,52,57]. Jamilian et al. (2019) reported that, compared to the vitamin D placebo plus probiotic placebo, the vitamin D plus probiotic decreased hs-CRP (β = −1.80 mg/L, *p* < 0.001) and MDA (β = −0.43 μmol/L, *p* = 0.01), and increased TAC (β = +97.77 mmol/L, *p* < 0.001) [52]. Compared to the vitamin D placebo plus probiotic (i.e., probiotic only), vitamin D plus probiotic increased GSH (β = +53.61 μmol/L, *p* = 0.04) and TAC (β = +63.26 mmol/L, *p* = 0.006). The probiotic alone decreased hs-CRP (β = −1.36 mg/L, *p* < 0.001) and MDA (β = −0.50 μmol/L, *p* = 0.005) compared to vitamin D placebo plus probiotic placebo group (i.e., neither vitamin D nor probiotic) [52]. In another RCT, Hajifaraji et al. (2018) and Dolatkah et al. (2015) reported that a probiotic decreased hs-CRP, TNF-⍺, and MDA by 9% (*p* = 0.019), 2% (*p* = 0.009), and 20% (*p* = 0.002), respectively, compared to the placebo [39,40]. An 18% increase in glutathione reductase (*p* = 0.047) and the preservation of erythrocyte GPx (−6% vs. −27%, *p* = 0.032) were also reported [39,40]. Changes in other antioxidant and inflammatory biomarkers were not noted [39,40]. In a third trial with probiotics, Jafarnejad et al. (2016) reported a decrease in hs-CRP (−14%, *p* = 0.03), interleukin-6 (IL-6, −10%, *p* = 0.04), and TNF-⍺ (−17%, *p* = 0.04) but not interleukin−10 or interferon gamma compared to the placebo group [57].

Two other RCTs supplemented synbiotics in women with GDM [54,55]. In the first RCT, Karamali et al. (2018) provided a synbiotic with inulin and reported reductions in hs-CRP (−24%, *p* = 0.004; adjusted change = −1.8 ± 0.6 mg/L, *p* = 0.002) and MDA (−4%, *p* = 0.02; adjusted change = −0.1 ± 0.1 μmol/L, *p* = 0.02) and elevations in GSH (+6%, *p* = 0.02; adjusted change = +31.3 ± 13.0 μmol/L, *p* = 0.01) and TAC (+7%, *p* = 0.009, adjusted change = +80.3 ± 19.9 mmol/L, *p* < 0.001) compared to the placebo group [54]. In the second RCT, Nabhani et al. (2018) reported that women receiving a synbiotic showed increases in TAC compared to the placebo group (+8%, *p* < 0.05) [55].

#### 3.2.4. Trace Elements

Three RCTs supplemented trace elements in women with GDM [51,58,60]. Most recently, Jamilian et al. (2019) reported that calcium, magnesium, and zinc co-supplemented with vitamin D decreased hs-CRP and MDA by 17% (*p* = 0.01) and 10% (*p* = 0.003), respectively, and increased TAC by 6% (*p* = 0.01) [51]. The changes in GSH and total nitrite were not significantly different compared to placebo group [51]. Prior to this trial, Karamali et al. (2016) supplemented zinc gluconate and reported decreases in hs-CRP (−3%, *p* = 0.03; adjusted change = −276.4 ± 407.5 ng/mL, *p* = 0.01) and MDA (−6%, *p* = 0.006). Further, TAC increased by 9% (*p* = 0.006; adjusted change = +51.9 ± 22.3 mmol/L, *p* = 0.03). Lastly, Asemi et al. (2015) supplemented selenium and reported decreased hs-CRP (−15%, *p* = 0.02; adjusted change = −881.2 ± 378.6 ng/mL) and increased GSH (+12%, *p* = 0.002; adjusted change = +57.7 ± 19.6 μmol/L, *p* < 0.001) [60]. The absolute change in TAC was not significant, but the adjusted change was significant (+70.8 ± 18.1 mmol/L, *p* = 0.02).

#### 3.2.5. Vitamin D

Five RCTs provided vitamin D as the sole dietary component in the supplement or as part of a combined supplement for co-supplementation in GDM [48,51,52,56,59]. The RCTs that co-supplemented vitamin D with other bioactive compounds are reported above in Section 3.2.2 Omega-3 (*n*-3) Fatty Acids [56], Section 3.2.3 Probiotics and Synbiotics [52], and Section 3.2.4 Trace Elements [51]. Zhang et al. (2016) reported that high, medium, and low doses of vitamin D alone until delivery increased GSH (+199, +158, and +102 µmol/L, respectively, vs. −28 µmol/L in the placebo group, *p* < 0.01) and TAC (+120, +89, and +49 mmol/L, respectively, vs. +12 mmol/L in the placebo group, *p* < 0.01) but did not affect hs-CRP [48]. Yazdchi et al. (2016) similarly reported that vitamin D alone did not reduce hs-CRP, but hs-CRP increased by 34% in the placebo group (*p* = 0.01; adjusted change as median [25th percentile−75th percentile] = +0.65 [0–1.40] mg/L, *p* = 0.02) [59].

## 4. Discussion

### 4.1. Observational Studies

Observational studies revealed lower adiponectin among women with GDM compared with healthy controls [44,46]. Adiponectin is a cardioprotective adipokine that dampens endogenous hepatic glucose production, improves insulin sensitivity, negatively correlates with body mass, and is often decreased in the presence of insulin resistance and/or diabetes mellitus [64,65]. Definitive conclusions about resistin and visfatin cannot be drawn from two studies, but these two biomarkers are relevant to GDM and deserve more attention [65]. Resistin is associated with obesity and insulin resistance and is increased or decreased in GDM [65]. Visfatin is another adipokine secreted by visceral body fat; the molecule is believed to be an insulin mimetic that exerts pro-inflammatory effects [65]. Whereas some studies have reported higher visfatin in GDM, other studies have reported lower concentrations [65].

Observational studies further provide evidence on the link between inflammation and GDM. Shivappa et al. (2019) demonstrated a positive association between an inflammatory diet before pregnancy and the development of GDM [41]. This study aligns with another case-control study with non-pregnant adults, where those consuming higher levels of inflammatory diets had nearly 19-fold greater odds of developing prediabetes compared to adults consuming lower levels of such diets [66]. Biomarkers of oxidative stress and inflammation, specifically hs-CRP [46] and MDA [45,47], were also elevated in GDM. However, in the study by Park et al. (2013), only women with overweight and GDM had elevated hs-CRP; women with normal weight and GDM had comparable hs-CRP to the healthy pregnant controls [46]. The importance of body mass in mediating inflammation in GDM is supported by two other studies that did not report elevations in hs-CRP or other biomarkers of inflammation in women with GDM [43,44]. McManus et al. [44] suggested that the lack of differences may be explained by the similar body mass between women with GDM the controls [44]. Obesity is a key mediator of inflammation [20,21], even in the absence of GDM, and may be the principal modulator of inflammatory biomarkers in GDM. This is of clinical relevance because women with overweight or obesity gain more gestational weight than do women with normal weight, exacerbating the negative impact of inflammation on maternal and fetal health.

Another possible modulator of inflammation in GDM is insulin resistance, which is linked to increased levels of hs-CRP, IL-6, and TNF-α [67,68]. In the study by Haidari et al. (2016), where no significant differences in inflammatory biomarkers were reported, women with GDM had similar insulin resistance as the control group, measured as the homeostatic model assessment of insulin resistance [43]. In addition to insulin resistance, a poor vitamin D status is implicated in GDM. Haidari et al. (2016) reported lower vitamin D in women with GDM and a negative correlation between 25(OH)D and hs-CRP [43]; McManus et al. (2014) also reported lower vitamin D in women with GDM but did not report significant correlations between 25(OH)D and biomarkers of inflammation [44]. Elsewhere, an inverse correlation between 25(OH)D and hs-CRP was identified in a population cohort of older adults [69], and a meta-analysis concluded, albeit cautiously, that vitamin D supplementation reduces hs-CRP in adults [70]. Collectively, these suggest that vitamin D may reduce inflammation. New RCTs should determine the extent to which improving insulin resistance and supplementing vitamin D improve inflammation in GDM.

### 4.2. RCTs—Diet Therapy

Hernandez et al. (2016) reported similar OxLDL between women with GDM who consumed a high complex carbohydrate/low-fat diet and women with GDM who consumed a low-carbohydrate/high-fat diet [63]. OxLDL is derived from circulating LDL and contains peroxides or products of their degradation [71]. The RCT by Hernandez et al. (2016) was the only one to measure OxLDL, and the trial was limited by its small sample size. The ability of the diet to reduce OxLDL is plausible, given its efficacy in reducing several other biomarkers of lipemia and inflammatory gene expression [63].

Asemi et al. (2013) evaluated inflammatory biomarkers associated with the DASH diet versus a control diet in GDM. The DASH diet emphasizes fruits, vegetables, whole grains, and lean meats and limits foods high in saturated fat and added sugar [72]. Asemi et al. (2013) reported increases in GSH and TAC with the DASH diet but non-significant changes in hs-CRP. A previous meta-analysis of six studies with non-pregnant adults reported that, when compared to other healthy diets, the DASH diet did not reduce hs-CRP [73]. A possible explanation for this observation is that reductions in hs-CRP are associated with confounders like weight loss rather than the DASH diet per se; in the study by Asemi et al. (2013), there were no differences in body mass or BMI between women with GDM on the DASH diet compared to those on the control diet. Certainly, adiposity is a major determinant of hs-CRP in GDM, but the effects of diets independent of adiposity need to be determined in future studies. The increase in TAC and GSH in this RCT could have been caused by the high intakes of antioxidant-rich fruits and vegetables and increased vitamin C in the DASH diet compared to the control diet [62].

Jamilian and Asemi (2015) compared a soy-protein-based diet to a control-protein-based diet in women with GDM and reported decreased MDA but not hs-CRP with soy [61]. A recent meta-analysis about soy and hs-CRP found that the evidence for soy improving hs-CRP among adults with a wide range of diseases is weak [74]. The inconsistent data on the topic are likely due to variations in the duration, sample size, and sample characteristics (biological sex, location, and disease status) of relevant RCTs [74]. Though the influence of soy on hs-CRP is controversial, soy may improve endogenous antioxidant defense systems. Fei et al. (2014) reported an increase in antioxidant enzymes such as catalase, GPx, and superoxide dismutase after participants consumed soybean oligosaccharides [49]. Also reported were an increase in adiponectin and a decrease in TBARS [49]. Endogenous TBARS are formed during lipid peroxidation and have been quantified in human tissues to determine the extent of oxidative stress [75]. In the same trial, insulin resistance may have improved as a result of increased adiponectin [49], an important mediator of the insulin signaling pathway [76].

### 4.3. RCTs—n-3 Fatty Acids

The antioxidant and anti-inflammatory effects of DHA and EPA in GDM are evidenced by three RCTs that suggest, collectively, that *n*-3 fatty acids reduced hs-CRP and MDA and increased GSH and TAC [50,53,56]. The antioxidant effects of *n*-3 fatty acids were previously demonstrated in adults with hypertriglyceridemia [77]. Moreover, a 2018 meta-analysis reported decreased biomarkers of inflammation and lipemia in patients with T2DM who ingested *n*-3 fatty acids, demonstrating that these dietary bioactive compounds may mitigate inflammation [78].

### 4.4. RCTs—Probiotic and Synbiotics

Probiotics, either alone or in conjunction with vitamin D, improved several biomarkers of inflammation, including hs-CRP, MDA, IL-6, and TNF-α in GDM [39,40,52,57]. Synbiotics also improved hs-CRP, GSH, and TAC [54,55]. The anti-inflammatory effects of co-supplementing vitamin D and bacterial strains found in probiotics and synbiotics have already been shown in children with allergic asthma, in whom vitamin D and *L. reuteri* for three months improved bronchial inflammation [79]. Supplementing a probiotic for three weeks in healthy adults reduced serum hs-CRP and the production of TNF-α in cultured peripheral blood mononuclear cells [80]. Additionally, a 2020 meta-analysis of seven RCTs in women with GDM concluded that probiotics reduced hs-CRP and MDA but did not affect GSH or nitric oxide [81]. In patients with T2DM and coronary artery disease, vitamin D alone has improved glycemia, GSH, hs-CRP, MDA, and nitric oxide [82]. Co-supplementation may improve inflammation in GDM primarily by attenuating oxidative stress, a key mediator of insulin resistance in GDM [83]. Strains of lactic acid bacteria (often found in both probiotics and synbiotics) scavenge reactive oxygen species, form chelates with metal ions, and inhibit oxidative enzymes [84]. Furthermore, probiotics are a source of short-chain fatty acids in the gut that likely modulate the microbiota to improve gut inflammation, insulin resistance, and weight control [85,86], all of which influence chronic, low-grade, systemic inflammation. Vitamin D may augment the anti-inflammatory effect by inhibiting pro-inflammatory genes involved in the production of inflammatory cytokines [50].

### 4.5. RCTs—Trace Elements

All three RCTs that supplemented trace elements in GDM reported decreased hs-CRP and increased TAC [51,58,60]. Jamilian et al. (2019) contended that magnesium, zinc, and vitamin D reduced hs-CRP by inhibiting the production of reactive oxygen species, the activity of the nuclear factor kappa light-chain enhancer of activated B cells, and peroxisome proliferator-activated receptor-alpha pathways [51]. The efficacy of supplementing selenium could be due to its promotion of the GPx-1 antioxidant defense system that functions to break down hydrogen peroxide, a source of oxidative stress [60,87]. In support of this, a meta-analysis reported that selenium improves GPx concentrations in addition to increasing TAC and decreasing MDA [88]. Levels of MDA and GPx are typically increased and decreased in GDM, respectively, and MDA correlates negatively with GPx [89]. The present review points to magnesium, selenium, zinc, and co-supplementation with vitamin D as a promising intervention for improving inflammation in GDM.

### 4.6. RCTs—Vitamin D

As previously mentioned, three RCTs co-supplemented vitamin D with other dietary bioactive compounds [51,52,56], and their effects were discussed above. Neither RCT that supplemented vitamin D alone in GDM reported a significant reduction in hs-CRP [48,59], but one trial showed an increase in TAC and GSH in a dose-dependent fashion [48]. Thus, vitamin D supplementation alone may be suitable for increasing antioxidant defenses, but it is not apparent that these effects reduce the important inflammatory marker hs-CRP as was observed with co-supplementation of vitamin D with other bioactive compounds [51,52,56]. Probable modulators of the efficacy of vitamin D supplementation include the severity of GDM; the type, dose, and duration of supplementation; and patients’ pre-supplementation metabolic variables, including vitamin D status and inflammatory biomarkers [59]. Thus, vitamin D may best act synergistically with other dietary compounds in reducing inflammation in GDM, and this needs further attention in future trials.

### 4.7. Strengths and Limitations

This paper has several strengths. First, the authors identified and evaluated observational studies and RCTs to address the lack of a comprehensive review on the associations of dietary bioactive compounds with maternal adiposity, antioxidant vitamin and mineral status, oxidative stress, and inflammation in GDM. Second, the authors utilized a structured literature search and selection process using the eligibility criteria. Third, all observational studies had at least one control group, and all RCTs had at least one control or placebo group. Additionally, all 17 of the clinical trials were randomized, thus reducing selection bias, and 14 of the 17 RCTs (82%) were double blinded, reducing experimenter bias. Fourth, the RCTs implemented a variety of interventions, including dietary patterns, *n*-3 fatty acids, probiotics, synbiotics, trace elements, and vitamins with bioactive roles in humans.

It is also worth noting a few limitations of the present review. First, our review is a semi-quantitative review that was not intended to be a systematic review or meta-analysis. This means that we did not implement the criteria of established systematic review methodologies (e.g., Cochrane Reviews), such as formal bias assessments. We also did not run statistical analyses on grouped data from separate studies, largely because the interventions consisted of disparate natural foods and/or dietary supplements. Conducting a systematic review and meta-analysis would have required a focus on a single intervention and outcome of interest, preventing us from giving readers an overview of many dietary bioactive compounds and biomarkers in GDM. In conducting the present review, our team agreed upon and followed a structured plan for searching, organizing, and reporting data from observational studies and RCTs (see Materials and Methods). Additionally, for the outcome measures of the RCTs, we calculated percentage differences between the treatment and control/placebo groups, hence why we describe our review as semi-quantitative. Ultimately, our review presents meaningful conclusions about the current literature on the effects of dietary bioactive compounds on maternal biomarkers of adiposity, antioxidant vitamin and mineral status, oxidative stress, and inflammation in GDM. Second, the literature search was conducted using only the three online databases Google Scholar, Medline, and PubMed. Though our chosen databases represent only a small number of available databases, they are major repositories of published health research. Third, this review presents only published observational studies and RCTs that were written in English. Fourth, while most of the observational studies and RCTs reported biomarkers of inflammation in GDM, only two observational studies and one RCT reported biomarkers of adiposity. This limitation highlights the need for investigations into the effects of different diets and their bioactive compounds on adiposity in GDM.

### 4.8. Future Directions

The biomarkers of adiposity, antioxidant vitamin and mineral status, oxidative stress, and inflammation evaluated in the present review are conventional variables often measured in women with GDM. The biomarkers are generally useful in providing insight into the endocrine activity of adipocytes, the degree to which biological antioxidant defenses are balanced with oxidative processes, and whether there is systemic inflammation [90,91]. However, the relationships of these biomarkers with the risk of GDM and related outcomes are not yet fully understood. Keeping this in mind, we acknowledge that the findings we report in this review inform readers of the effects of dietary interventions on these biomarkers in GDM but do not provide data for precisely predicting the risk or prognosis of GDM. Therefore, we recommend that investigators conduct new systematic reviews and meta-analyses on specific interventions with dietary bioactive compounds to elucidate their effects (quantitatively) on the risk of GDM, adiposity, and chronic oxidative stress and inflammation. When there are insufficient data to conduct a well-powered meta-analysis on a specific dietary intervention in GDM, new trials will be needed to address the gap.

In addition to conducting meta-analyses on the conventional biomarkers, a future focus should be placed on metabolomics and proteomics research in GDM. These emerging fields may enable health professionals to identify and quantify maternal and fetal metabolites and proteins that are clinically relevant to GDM. Metabolomics research is attempting to characterize the metabolic profile of women with GDM compared to women without the disease and how treatment modality affects the profile. In a recent report (2020), levels of certain lipid, amino acid, and acetylated glycoprotein metabolites were shown to be greater in women with overweight or obesity and GDM and to be contingent on treatment type (diet alone vs. medication) [92]. Ascertaining the metabolic profiles of women at risk for or diagnosed with overt GDM will further shape precision medicine in which complex metabolic profiles are used to correctly identify at-risk women, aid in diagnosis, predict the efficacy of therapies, and individualize treatment. In addition to metabolomics research, ongoing proteomics research in GDM is attempting to discern proteins from various maternal tissues that will enable early and accurate predictions of risk and complications as well as precise diagnoses [93]. In summary, it is highly likely that research in metabolomics and proteomics will generate profound advancements in treating GDM.

## 5. Conclusions

**Figure 2:**
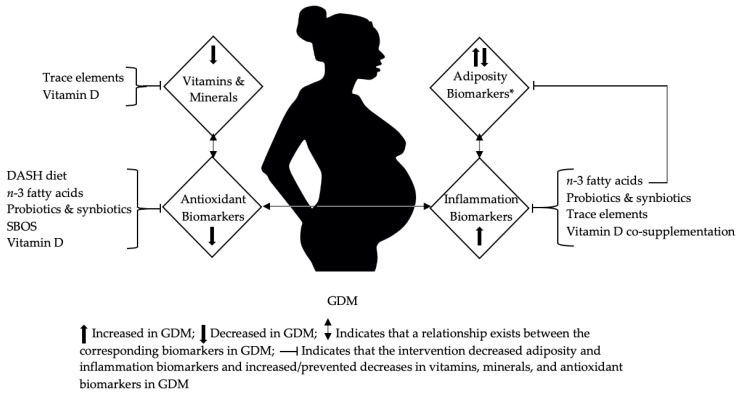
Effects of supplementing vitamins, minerals, and other dietary bioactive compounds on adiposity, vitamin and mineral status, antioxidant biomarkers, and inflammation in gestational diabetes mellitus (GDM). DASH: the Dietary Approaches to Stop Hypertension; SBOS: soybean oligosaccharides. * Only one RCT reported data on adiposity biomarkers (Supplementing SBOS increased adiponectin, Fei et al., 2014).

The present review is a semi-quantitative analysis of 24 studies that serves to update readers on the associations of dietary bioactive compounds with maternal biomarkers of adiposity, antioxidant vitamin and mineral status, oxidative stress, and inflammation in GDM. We report several noteworthy findings. Women with GDM consume more energy, carbohydrates, and fats but smaller amounts of antioxidant vitamins and minerals (vitamin E, selenium, and zinc) than healthy pregnant women. This dietary pattern is undesirable because it promotes maternal obesity, oxidative stress, and inflammation. Data also reveal that a pro-inflammatory diet in the year preceding conception is associated with double the risk of GDM compared to a low-inflammatory diet. Women who develop GDM tend to have lower blood levels of vitamins A, C, and D and adiponectin but a higher level of MDA. In aggregate, the RCTs reveal several promising diets containing natural foods (three studies) and many dietary supplements (14 studies) that contain dietary bioactive compounds shown to improve biomarkers of inflammation in women with GDM (Figure 2). The DASH diet and soybean oligosaccharides may increase antioxidant enzyme concentrations. Supplementing *n*-3 fatty acids, probiotics, synbiotics, and trace elements also enhances blood antioxidant defenses while reducing inflammation, indicated by lower levels of hs-CRP and MDA. Providing vitamin D alone does not seem to confer the same benefits.

## Figures and Tables

**Table 1:** Observational studies on maternal biomarkers of adiposity, antioxidant vitamin and mineral status, oxidative stress, and inflammation in gestational diabetes mellitus.

Authors, Year (Country)	Study Design	Participants	Assessment of Nutritional Status	Vitamins and Minerals	Anthropometrics and/or Body Composition	Adiposity	Oxidative Stress and Inflammation
Shivappa et al., 2019 (Iran) [41]	Case Control	Women with GDM (*n* = 121) Age: 29.6 ± 4.5 years; BMI: 27.3 ± 3.8 kg/m^2^ Healthy pregnant CON (*n* = 266) Age: 29.8 ± 4.3 years; BMI: 24.6 ± 3.3 kg/m^2^	Dietary inflammatory index (DII) FFQ (147 items) Blood samples	NR	NR	NR	↑ odds of GDM with higher DII scores
Parast et al., 2017 (Iran) [42]	Case Control	Women with GDM (*n* = 40) Age: 29.4 ± 4.9 years; BMI: 25.2 ± 2.3 kg/m^2^ Healthy pregnant CON (*n* = 40) Age: 28.9 ± 5.2 years; BMI: 24.5 ± 2.8 kg/m^2^	FFQ (168 items) Blood samples	GDM: intake of ↓ vit E, Se, Zn NS—β-carotene, vit C ↓ odds of GDM with ↑ intake of vit E and Zn	NR	NR	↓ TAC in GDM vs. CON ↓ odds of GDM with ↑ TAC
Haidari et al., 2016 (Iran) [43]	Cross Sectional	Women with GDM (*n* = 45) Age: 29.3 ± 4.3 years; BMI: 25.7 ± 3.1 kg/m^2^ Healthy pregnant CON (*n* = 45) Age: 27.5 ± 4.9 years; BMI: 24.3 ± 3.0 kg/m^2^	No dietary data Blood samples	GDM: ↓ 25(OH)D	GDM and CON: 25(OH)D negatively correlated with pre-pregnancy BMI	NR	GDM and CON: 25(OH)D negatively correlated with hs-CRP NS—hs-CRP, TNF-α
McManus et al., 2014 (Canada) [44]	Case Control	Women with GDM (*n* = 36) Age: 31.6 ± 5.0 years; BMI: 28.7 ± 5.5 kg/m^2^ Healthy pregnant CON (*n* = 37) Age: 30.2 ± 4.1 years; BMI: 27.2 ± 7.2 kg/m^2^	No dietary data Blood samples	GDM: ↓ 25(OH)D	NR	GDM: ↓ adiponectin, resistin NS—leptin	NS—hs-CRP, IL-6, IL-8, MCP-1, PAI-1, TNF-α
Javadian et al., 2014 (Iran) [45]	Case Control	Women with GDM (*n* = 52) Age: 31.2 ± 6.7 years; BMI: 26.9 ± 3.8 kg/m^2^ Healthy pregnant CON (*n* = 50) Age: 28.9 ± 6.7 years; BMI: 25.7 ± 4.4 kg/m^2^	No dietary data Blood samples	GDM: ↑ ferritin	NR	NR	GDM: ↑ MDA
Park et al., 2013 (South Korea) [46]	Case Control	Women with normal weight and GDM (*n* = 98) Women with overweight and GDM (*n* = 117) Healthy pregnant CON with normal weight (*n* = 395) Healthy pregnant CON with overweight (*n* = 136) Combined age NR; Combined BMI: 23.29 ± 3.59 kg/m^2^	Interviewer-administered FFQ 24 h recall Blood samples	NR	NR	Groups with overweight vs. groups with normal weight: ↑ leptin, adipsin GDM + overweight vs. other groups: ↓ visfatin GDM vs. CON: ↓ adiponectin	GDM + overweight vs. other groups: ↑ CRP
Suhail et al., 2010 (India) [47]	Cross Sectional	Women with GDM (*n* = 23) Age: 28.0 ± 4.0 years; BMI at delivery: 21.0 ± 2.4 kg/m^2^ Healthy pregnant CON (*n* = 23) Age: 27.0 ± 4.0 years; BMI at delivery: 20.1 ± 1/4 kg/m^2^ Non-pregnant CON (*n* = 23) Age: 26.0 ± 5.0 years; BMI at delivery: 20.4 ± 2.6 kg/m^2^	No dietary data Blood samples	GDM vs. healthy, pregnant CON: ↓ vit C, vit E GDM and healthy, pregnant CON vs. non-pregnant CON: ↓ vit A	NR	NR	GDM and healthy, pregnant CON vs. non-pregnant CON: ↑ MDA GDM vs. healthy, pregnant CON: ↑ MDA

Up (↑) and down (↓) arrows indicate an increase and decrease, respectively. 25(OH)D: 25-hydroxyvitamin D; β-carotene: beta-carotene; BMI: body mass index; CON: control; DII: dietary inflammatory index; FFQ: food frequency questionnaire; GDM: gestational diabetes mellitus; hs-CRP: high-sensitivity C-reactive protein; IL-6: interleukin-6; IL-8: interleukin-8; kg: kilograms; m: meters; MCP-1: monocyte chemoattractant protein-1; MDA: malondialdehyde; *n* = sample size; NR: not reported; NS: not significantly different from the control group(s); PAI-1: plasminogen activator inhibitor-1; Se: selenium; TAC: total antioxidant capacity; TNF-α: tumor necrosis factor-α; vit A: vitamin A; vit C: vitamin C; vit E: vitamin E; Zn: zinc.

**Table 2:** Randomized controlled trials on the effects of dietary bioactive compounds on maternal biomarkers of adiposity, antioxidant vitamin and mineral status, oxidative stress, and inflammation in gestational diabetes mellitus.

Authors, Year (Country)	Trial Design	Participants	Dietary Intervention and Duration ^1^	Antioxidant Vitamins and Minerals	Anthropometrics and/or Body Composition	Adiposity	Oxidative Stress and Inflammation
Jamilian et al., 2020 (Iran) [50]	Double-blind RCT	Women with GDM (*n* = 51) Age: 29.0 ± 4.6 years; BMI: 28.1 ± 4.5 kg/m^2^	2000 mg *n*-3 FA from flaxseed oil (800 mg ALA), daily Capsules, 6 weeks	NR	NS—height, mass, BMI	NR	↓ hs-CRP, MDA ↑ GSH, total nitrite
Jamilian et al., 2019 (Iran) [51]	Double-blind RCT	Women with GDM (*n* = 60) Age: 28.4 ± 4.1 years; BMI: ± 25.6 3.1 kg/m^2^	200 mg Mg + 8 mg Zn + 800 mg Ca + 400 IU vit D, daily Tablets, 6 weeks	↑ Mg, Zn, Ca, 25(OH)D	NS—height, mass, BMI	NR	↓ hs-CRP, MDA ↑ TAC NS—total nitrite, GSH
Jamilian et al., 2019 (Iran) [52]	Double-blind RCT	Women with GDM (*n* = 87) Age: 30.0 ± 5.4 years; BMI: 27.2 ± 4.2 kg/m^2^	G1: 50,000 IU vit D (biweekly) + probiotic (daily) G2: Probiotic (daily) + vit D PBO (biweekly) G3: Vit D PBO + probiotic PBO Capsules, 6 weeks	G1 vs. G2 and PBO; G2 vs. PBO: ↑ 25(OH)D	NS—height, mass, BMI	NR	G1 vs. PBO; G2 vs. PBO: ↓ hs-CRP, MDA G1 vs. G2 and PBO: ↑ TAC G1 vs. G2: ↑ GSH
Hajifaraji et al., 2018 (Iran) [39] and Dolatkhah et al., 2015 (Iran) [40]	Double-blind RCT	Women with GDM (*n* = 56) Age: 27.3 ± 5.8 years; BMI: 30.7 ± 3.7 kg/m^2^	Probiotic, daily Capsules, 8 weeks	NR	↓ body mass gained in the last two and four weeks of the study	NR	↓ hs-CRP, lnTNF-α, MDA ↑ erythrocyte GPx, GSHR NS—erythrocyte SOD, IL-6, TAC, uric acid
Jamilian et al., 2018 (Iran) [53]	Double-blind RCT	Women with GDM (*n* = 40) Age: 30.7 ± 3.1 years; BMI: 27.5 ± 2.9 kg/m^2^	2000 mg fish oil (360 mg EPA and 240 mg DHA), daily Capsules, 6 weeks	NR	NS—height, mass, BMI	NR	↓ hs-CRP
Karamali et al., 2018 (Iran) [54]	Double-blind RCT	Women with GDM (*n* = 60) Age: 26.7 ± 4.7 years; BMI: 28.5 ± 3.5 kg/m^2^	Synbiotic + 800 mg inulin, daily Capsules, 6 weeks	NR	NS—height, mass, BMI	NR	↓ hs-CRP, MDA ↑ GSH, TAC NS—NO
Nabhani et al., 2018 (Iran) [55]	Double-blind RCT	Women with GDM (*n* = 90) Age: 29.9 ± 5.7 years; BMI: 27.3 ± 4.5 kg/m^2^	Synbiotic Capsules, 6 weeks	NR	NS—body mass gained, BMI, hip circumference	NR	↑ TAC
Razavi et al., 2017 (Iran) [56]	Double-blind RCT	Women with GDM (*n* = 120) Age: 29.7 ± 4.0 years; BMI: 29.0 ± 3.7 kg/m^2^	G1: 2000 mg *n*-3 FA (360 mg EPA, 240mg DHA, daily) + vit D PBO (biweekly) G2: *n*-3 FA PBO + 50,000 IU vit D (biweekly) G3: 2000 mg *n*-3 FA (daily) + 50,000 IU vit D (biweekly) G4: *n*-3 PBO (daily) + vit D PBO (biweekly) Capsules, 6 weeks	↑ 25(OH)D (G3)	NS—height, mass, BMI	NR	G3: ↓ hs-CRP, MDA G3: ↑ GSH, NO, TAC
Hernandez et al., 2016 (USA) [63]	RCT	Women with GDM (*n* = 12) Age: 29.0 ± 1.8 years; BMI: 33.9 ± 1.5 kg/m^2^	CHOICE diet vs. LC/CONV diet 6 weeks	NR	NS—mass, BMI	NR	NS—OxLDL
Jafarnejad et al., 2016 (Iran) [57]	Double-blind RCT	Women with GDM (*n* = 72) Age: 32.2 ± 3.6 years; BMI: 27.1 ± 2.9 kg/m^2^	Probiotic mixture, daily Capsules, 8 weeks	NR	NS—height, mass, BMI	NR	↓ hs-CRP, IL-6, TNF-α NS—IFN-γ, IL-10
Karamali et al., 2016 (Iran) [58]	Double-blind RCT	Women with GDM (*n* = 50) Age: 29.6 ± 4.4 years; BMI: 28.2 ± 3.6 kg/m^2^	233 mg Zn gluconate (30 mg zinc), daily Tablets, 6 weeks	NS—Zn	NS—height, mass, BMI	NR	↓ hs-CRP ↑ TAC NS—GSH, NO, MDA
Yazdchi et al., 2016 (Iran) [59]	Double-blind RCT	Women with GDM (*n* = 72) Age: 31.9 ± 4.0 years; BMI: 31.5 ± 3.7 kg/m^2^	50,000 IU vit D (biweekly) Capsules, 8 weeks	↑ 25(OH)D	NS—height, mass, BMI	NR	NS—hs-CRP
Zhang et al., 2016 (China) [48]	Double-blind RCT	Women with GDM (*n* = 133) Age: 29.9 ± 4.8 years; BMI: 30.9 ± 4.0 kg/m^2^	G1: 200 IU vit D (daily) G2: 2000 IU vit D (daily, 25 days, total of 50,000 IU) G3: 4000 IU vit D (daily, 12.5 days, total of 50,000 IU) Capsules, enrollment through delivery	NR	NR	NR	G1, G2, and G3: ↑ GSH, TAC NS—hs-CRP
Asemi et al., 2015 (Iran) [60]	Double-blind RCT	Women with GDM (*n* = 70) Age: 28.6 ± 4.6 years; BMI: 27.9 ± 4.0 kg/m^2^	200 μg Se (daily) Capsules, 6 weeks	NR	NS—height, mass, BMI	NR	↓ hs-CRP ↑ GSH, TAC NS—MDA, NO
Jamilian et al., 2015 (Iran) [61]	RCT	Women with GDM (*n* = 68) Age: 28.8 ± 4.4 years; BMI: 28.7 ± 4.3 kg/m^2^	Diet with 35% soy, 35% animal, and 30% other plant protein vs. CON diet with 70% animal and 30% other plant protein 0.8 g protein/kg body mass in each diet 6 weeks	NR	NS—height, mass, BMI	NR	CON vs. Soy (Between): ↑ MDA NS—GSH, hs-CRP, NO, TAC
Fei et al., 2014 (China) [49]	RCT	Women with GDM (*n* = 97) Age: > 20 years; BMI: NR	10 g soybean oligosaccharides 200–300 mL water (daily) Orally, 8 weeks	NR	NR	↑ adiponectin	↑ SOD, CAT, GPx ↓ TBARS
Asemi et al., 2013 (Iran) [62]	Double-blind RCT	Women with GDM (*n* = 32) Age: 28.7 ± 5.5 years; BMI: 30.0 ± 3.9 kg/m^2^	DASH diet 4 weeks	NR	NS—height, mass, BMI	NR	↑ GSH, TAC NS—hs-CRP

Up (↑) and down (↓) arrows indicate an increase and decrease, respectively. ^1^ Reported dosage reflects the total daily quantity of dietary bioactive compounds ingested (in some cases, ingested across the day). 25(OH)D: 25-hydroxyvitamin D; ALA: alpha-linolenic acid; BMI: body mass index; Ca: calcium; CAT: catalase; CHOICE: higher-complex carbohydrate/lower-fat diet; CON: control group; DASH: Dietary Approaches to Stop Hypertension; DHA: docosapentanoic acid; EPA: eicosapentanoic acid; g: grams; G1: group 1 in the trial; G2: group 2 in the trial; G3: group 3 in the trial; GDM: gestational diabetes mellitus; GPx: glutathione peroxidase; GSH: reduced glutathione; GSHR: glutathione reductase; hs-CRP: high-sensitivity C-reactive protein; IFN-γ: interferon-gamma; IL-10: interleukin-10; IL-6: interleukin-6; IU; international units; kg: kilograms; LC/CONV: low-carbohydrate/higher-fat diet; lnTNF-α: logarithmically transformed tumor necrosis factor-α; m: meters; MDA: malondialdehyde; Mg: magnesium; mg: milligrams; mL: milliliters; *n* = sample size; *n*-3 FA: omega-3 fatty acids; NO: nitric oxide; NR: not reported; NS: not significantly different from the control/placebo group(s); OxLDL: oxidized low-density lipoprotein; PBO: placebo group; RCT: randomized controlled trial; Se: selenium; SOD: superoxide dismutase; TAC: total antioxidant capacity; TBARS: thiobarbituric acid reactive substances; vit D: vitamin D; Zn: zinc.
